# Reply to letters to the editor entitled “Bias, the unfinished symphony” and “Bias estimation for Sigma metric calculation: arithmetic mean versus quadratic mean”

**DOI:** 10.11613/BM.2023.020401

**Published:** 2023-04-15

**Authors:** Murat Keleş

**Affiliations:** Alanya Alaaddin Keykubat University, Alanya Education and Research Hospital, Department of Biochemistry, Antalya, Turkey

**Keywords:** biochemistry, biological variation, quality control

## To the editor,

I would like to thank Coşkun and Ercan for their interest in my paper and their constructive contributions to the subject (*1*, *2*). I want to clarify the suggestions and criticisms made by Coşkun and Ercan regarding my paper.

To start with the “Due to random error, using a single measurement result is not adequate to calculate bias and at least a duplicate measurement is necessary” criticism that Coşkun stated in his paper (*2*). In our laboratory, monthly routine external quality control (EQC) practices are applied as “It is recommended to run the External Quality Control Samples (as repetition) once.” specified in the insert of the Turkish Association of Clinical Biochemistry Specialists (KBUDEK) EQC material and under the principles defined in the CLSI GP27-A2 document: “Some laboratories may improperly test proficiency testing (PT) samples differently from patient samples, by repeat testing of PT samples when patient samples are tested only once, or by having a specific analyst test PT samples rather than rotating PT testing among all the personnel who perform patient testing. These practices defeat the utility of proficiency testing and rob the laboratory of important information about the quality status of the laboratory’s procedures and processes.” (*3*, *4*). Although the suggestion made by Coşkun for the calculation of bias is statistically meaningful, it is incompatible with routine laboratory practices. The calculation made in this way will both create question marks in the EQC efficiency and increase the costs at a very high rate. In addition, to minimize the effect of random errors, unacceptable results were excluded according to the EQC reports, and long-term process evaluation was made by using the EQC results of a twelve-month cycle. Considering these pluses and minuses, the average bias was used in the study. Ercan’s suggestion about the quadratic mean calculation of bias is generally the bias calculation method used in Nordtest measurement uncertainty studies. However, it was not applied because found not to be methodologically appropriate for our study. As for Coşkun’s criticism that the bias used in the traditional six sigma formula in our study was treated as a linear component and gave false low results and the formula was wrong; the observed method bias is subtracted from the tolerance limit permissible total error (pTE) because it narrows the region for acceptable performance. Bias accounts for the lack of “centerness” of the production distribution and is completely consistent with the industrial concept of process capability index (Cpk) (*5*). In line with the above information, I preferred the traditional six sigma calculation in the study since it is thought that it is not correct to consider the bias used in the sigma calculation as a uniform distribution, it only takes into account the shift in the center of the normal distribution compatibly to Cpk. When Coşkun’s “All these instruments are high-tech instruments, and their actual quality level is higher than the sigma metric (SM) calculated using this equation” criticism is examined; the first part of our two-stage study has already been based on the manufacturer’s reagent insert data, and the precision and bias calculations are based on the studies done by the manufacturer. Even in this case, it has been observed that some analytes do not meet the biological variation (BV) goals. Although it is thought that bias should be included in the sigma calculation, in order to better understand the results, when the Sigma_analyser_ performance was re-evaluated by neglecting the bias (SM = TEa/CV) in our study; it was observed that five analytes showed unacceptable performance similar to the traditional sigma calculation (SM = TEa-Bias/CV) in 10 out of a total of 42 goals at both two levels according to the BV goals. The only difference with the traditional sigma calculation was that creatinine level 1 showed borderline acceptable performance. As a result, I think that the problem is caused by the disproportion between goals and device performance rather than the method used in six sigma calculations. It will be easier for us to reach a solution when we determine the main source of the problem correctly.
